# Single-Port Versus Reduced-Port (1 + 1) Robotic Myomectomy and Hysterectomy

**DOI:** 10.3390/jcm13216563

**Published:** 2024-10-31

**Authors:** So Young Lee, Sa Ra Lee, Jae Yen Song

**Affiliations:** 1 Department of Obstetrics and Gynecology, Seoul Asan Medical Center, University of Ulsan College of Medicine, 88, Olympic-ro 43-gil, Songpa-gu, Seoul 05505, Republic of Korea; 2 Department of Obstetrics & Gynecology, College of Medicine, The Catholic University of Korea, 222 Banpo-daero, Seocho-gu, Seoul 06591, Republic of Korea

**Keywords:** robot-assisted laparoscopy, single-port surgery, reduced-port surgery

## Abstract

**Background**: We aimed to investigate the reduced-port (RP) robotic surgery as an alternative to the single-port (SP) robotic surgery by first comparing the surgical outcomes between the two groups. **Methods:** A total of 184 patients who underwent SP robotic myomectomy (SP-RM, *n* = 94), RP robotic myomectomy (RP-RM, n = 38), SP robotic hysterectomy (SP-RH, n = 33), or RP robotic hysterectomy (RP-RH, n = 19) from October 2020 to April 2022 were analyzed. An 8 mm skin incision 8–10 cm away from the umbilical incision was made for the RP robotic surgery. **Results:** Of the total of 184 patients, 166 (90.2%) underwent surgery for leiomyoma followed by adenomyosis (n = 10, 5.44%). None of the cases were converted to laparotomy or added additional ports during surgery. Demographic characteristics and surgical outcomes were comparable between RP-RM and SP-RM and between RP-H and SP-H, respectively. However, more leiomyoma were removed in the RP-RM group than the SP-RM group [median 4.2 (range 1–21) vs. median 2.7 (range 1–11), *p* = 0.009]. The total operating time was longer in the SP-RH group than the RP-RH group (159.8 ± 55.0 min vs. 113.6 ± 24.6 min, *p* = 0.001). **Conclusions:** RP robotic surgery is a feasible and effective surgical option for myomectomy and hysterectomy with cosmetic benefits compared to conventional multiport robotic surgery. In conclusion, for surgeons who cannot use the da Vinci SP, RP robotic surgery using the da Vinci^®^ Xi or Si systems may be an alternative option.

## 1. Introduction

Minimally invasive surgery is the gold standard operation option in the treatment of most gynecologic tumors and single-port laparoscopic surgery has expand the indications for cosmetic benefits and patient satisfaction [1,2]. However, single-port laparoscopic surgery is challenged by some limitations, especially in terms of collisions between instruments causing limited instrument movement and poor ergonomics [3,4,5]. The technology of a single-port robotic system enables surgeons to overcome the disadvantages of single-port laparoscopic surgery [6,7].

Since the first report of robotic single-site surgery (RSSS) in the gynecologic field in 2010, the approach has acquired a wide range of applications in gynecological procedures [8,9,10,11,12,13]. However, RSSS still has some technical limitations including difficulties in handling the semi-rigid instruments and the difficult of suturing using instruments with weak power causing jerky surgical movement. The crowding and conflicts between the camera and the instruments are not completely resolved. The limited number of instruments with an endowrist function are an obstacle to broadening the applications of RSSS [10,14,15].

To achieve both satisfactory cosmetic results and overcome the surgical limitations of RSSS, we used an alternative method, a reduced-port (1 + 1) robotic surgery method using conventional rigid multiport robotic instruments. This method needs only two skin incisions, one 2.5 cm intraumbilical incision for a multichannel laparoscopic single port and one additional 8 mm skin incision 8–10 cm to the right side of the umbilicus for a multiport robotic cannula. The robotic camera and one multiport robotic cannula are inserted through a multichannel laparoscopic single port and the other multiport robotic instrument is inserted into the additional robotic cannula.

In June 2018, the da Vinci^®^ SP system, a fourth-generation model, was introduced specifically for single-port surgery. Through technological advances, all instruments for the da Vinci^®^ SP system have two joints. It was therefore hypothesized that by combining a variety of instruments with the new da Vinci^®^ SP surgical system that had adequate articulation, appropriate power, and less crowding, surgeons might be able to overcome the RSSS restrictions encountered when using the da Vinci^®^ Xi or Si systems.

However, the availability of the da Vinci^®^ SP system is still limited in most hospitals because the most distributed types of da Vinci^®^ robotic system are the da Vinci^®^ Xi or Si systems. Surgeons also can select RSSS for the same cosmetic results; however, RSSS with semi-rigid instruments has limited indications due to the disadvantages described above. Myomectomy and hysterectomy of a huge uterus do not have good indications for RSSS, except for very skillful experienced surgeons. Therefore, surgeons can select the reduced-port (1 + 1) method using multiport robotic instruments to minimize scarring by using the da Vinci^®^ Xi or Si systems. This reduced-port method can be a useful alternative option for single-port robotic surgeries using the da Vinci^®^ SP and RSSS.

In this study, we aimed to first compare the surgical outcomes between single-port robotic surgery using da Vinci SP system and reduced-port (1 + 1) robotic surgery using the da Vinci^®^ Xi or Si systems for myomectomy and hysterectomy.

## 2. Materials and Methods

### 2.1. Study Design and Patients

A retrospective chart review was performed. We analyzed the medical charts of a total of 184 patients who underwent single-port robotic myomectomy (SP-RM, n = 94), reduced-port robotic myomectomy (RP-RM, n = 38), single-port robotic hysterectomy (SP-RH, n = 33), or reduced-port robotic hysterectomy (RP-RH, n = 19) from October 2020 to April 2022. The choice of SP or RP surgery was made after a comprehensive discussion with the patient, taking into account not only the surgical scar but also the size, type, and number of myomas. The preference of the patients and surgeons and the availability of each robotic system were also considered. This study was approved by the Asan Medical Center Institutional Review Board (approval No. 2021-0808).

Medical records including age, gravidity, parity, BMI, history of previous cesarean section, and/or pelvic surgery were review and collected for analysis. The indications of myomectomy were symptomatic fibroids causing excessive menstrual bleeding, pelvic pain or pressure, and adversely affecting reproductive outcomes by deviating the uterine axis. For myomectomy, myomas were categorized according to the International Federation of Gynecology and Obstetrics (FIGO) types and classified into submucosal (0, 1, 2), intramural (FIGO types 3, 4, 5), subserosal (FIGO types 6, 7), or others such as intra-ligamentary or cervical. The following data were collected to assess surgical and perioperative outcomes: the maximum size and number of myomas, the total operation time (skin incision to closure), and conversion to laparotomy were the primary outcomes; and secondary outcomes are the weight of the removed myomas (as weighed during the pathologic examination), concomitant surgeries, estimated blood loss (EBL), blood transfusion, postoperative Hb level, length of hospital stay, postoperative fever within 48 h, and any complications during or after surgery.

### 2.2. Surgical Procedures

Under general anesthesia, the patients were placed in the dorsal lithotomy position. In order to perform SP-RM or SP-RH using the da Vinci^®^ SP system, we established a 27~30 mm transumbilical skin incision and opened the fascia layer with the open Hassel technique. Then, we inserted the multichannel single port specifically designed for the da Vinci^®^ SP system such as Uni-port (Dalim, Seoul, Republic of Korea), Gloveport (Meditech inframed, Seoul, Republic of Korea), or Lapsingle Vision (Sejong medical, Paju, Gyeonggi-do, Republic of Korea). These ports have four lumens, one is for the multichannel SP cannula, which has one lumen for the SP camera and three lumens for the SP robotic instruments and three 5–10 mm additional pre-placed ports. Monopolar curved scissors for the right (3 o’clock) robotic arm, fenestrated bipolar forceps for the left (9 o’clock) robotic arm, and a needle driver for the centrally positioned (6 o’clock) robotic arm were used.

**Figure 1:**
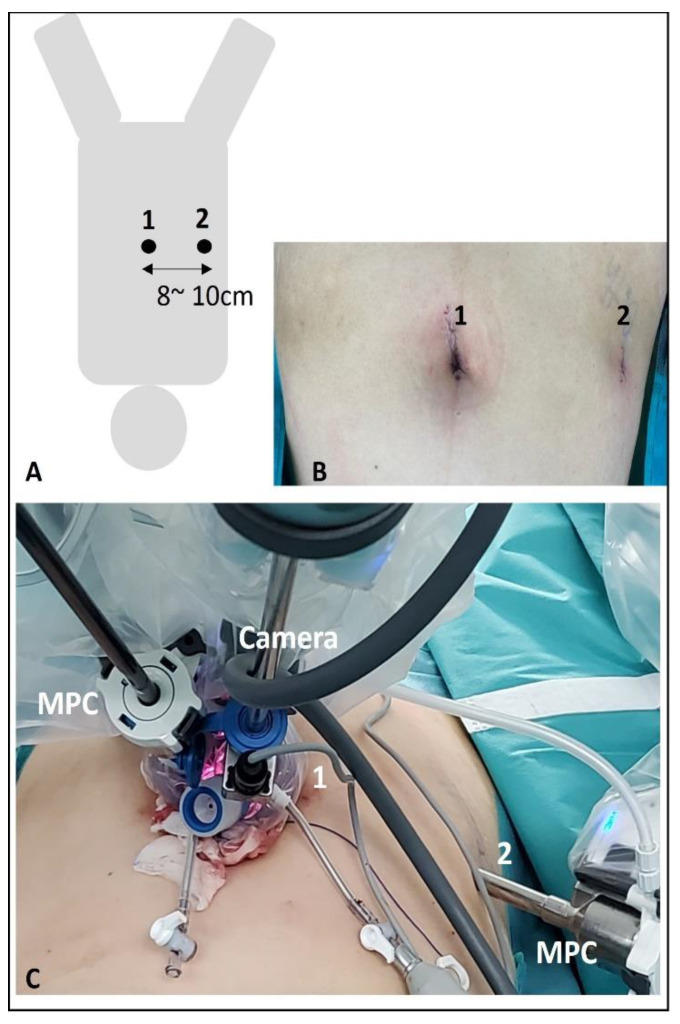
Reduced-port (1 + 1) robotic surgery using the da Vinci^®^ Si or Xi system. (**A**,**B**) Two skin incisions, a transumbilical incision about 2.5 cm in length (1) and an 8 mm incision (2) about 8–10 cm apart from the umbilicus along the flank line. (**C**) A multichannel single port designed for laparoscopic surgery was inserted in the transumbilical incision (1). An 8.5 mm cannula (Intuitive Surgical) for the camera was inserted into the lumen of the single port toward the center of the umbilicus incision, and an 8 mm straight multiport cannula (MPC) (Intuitive Surgical) for the left robotic arm (9 o’clock) was inserted into the lumen located on the left. To equip the right robotic (3 o’clock) arm, an 8 mm straight MPC was inserted along on the right abdomen with an incision a man’s fist apart from the umbilicus along the flank line (2).

The reduced-port approaches were begun by making a transumbilical incision about 2.5 cm in length, through which the multichannel single port designed for the laparoscopic surgery was inserted. This multichannel cannula compromises four lumens for instrumentation. An 8.5 mm cannula (Intuitive Surgical) was inserted into the lumen of the single port toward the center of the umbilicus incision, and an 8 mm straight cannula (Intuitive Surgical) for the left robotic arm (9 o’clock) was inserted into the lumen located on the left. To equip the right robotic (3 o’clock) arm, an 8 mm straight cannula was inserted into the right abdomen with an incision a man’s fist apart from the umbilicus along the flank line (Figure 1).

In the case of reduced-port surgery, monopolar scissors or a needle driver for the right robotic arm and fenestrated bipolar forceps or a vessel sealer for the left robotic arm were used. For SP-RM and RP-RM, a RUMI Arch™ (CooperSurgical Inc., Trumbull, CT, USA) or ZUMI (CooperSurgical Inc., Trumbull, CT, USA) uterine manipulator was placed. Myomas were retrieved from the uterus with the locking suture on myoma (LSOM) technique without the need for tenaculum forceps or laparoscopic tenaculum forceps in the usual manner [16]. A brief description of the LSOM technique follows. Upon exposure of the myoma after the incision on the uterine serosa, a locking suture with 1-0 V-Loc was made on the uterine myoma, and the myoma traction was easily performed by grasping the thread with the fenestrated bipolar forceps, needle holder, or laparoscopic atraumatic forceps. Whenever the dissection between the myoma and myometrium was advanced, the next locking suture was made on the additionally exposed myoma tissue at the nearest position to the dissection plane, and this process was repeated. This step-by-step locking suture offered an easier enucleation of the myoma than using the laparoscopic Tenaculum forceps. The retrieved myomas were easily removed by simply grasping the thread with the laparoscopic atraumatic forceps or laparoscopic needle holder and were extracted by manual morcellation using a scalpel within a bag, or sometimes in an uncontained state through the existing umbilical incision. The uterine myometrium was sutured layer by layer in two or three layers using a continuous running suture technique with absorbable barbed suture materials, including 1-0 V-Loc™ suture (Covidien, Mansfield, MA, USA) or 1-0 Monofix PDO (Samyang, Daejeon, Republic of Korea) and the uterine serosa was sutured with 1-0 Quill™ SRS bidirectional barbed suture (Angiotech Pharmaceuticals Inc., Vancouver, BC, Canada) in a baseball suture technique. The retrieved myomas were removed by scalpel morcellation using an endopouch. For SP-RH and RP-RH, a Uterine PositionORTM (Symmetry surgical Inc., Antioch, TN, USA) uterine manipulator was placed. Both the round and ovarian or infundibulopelvic ligaments were ligated using an electrosurgical bipolar vessel sealing device depending on whether the adnexa was removed. Subsequently, the anterior and posterior aspects of the broad ligament were dissected to the level of the uterine vessels. Next, vesicovaginal and rectovaginal space dissection and anterior and posterior colpotomy were performed with the help of an assistant pushing the manipulator. After anterior colpotomy, with the cervical cap exposed and the anterior vaginal cuff almost resected, the uterine vessels were sutured using 1-0 V-Loc™. After circular colpotomy was completed, the uterus was detached from the vault, and the uterine manipulator was removed. If the size of the uterus was small or the pubic arch was wide, the uterus was delivered through the vagina. If not, the uterus was removed through the umbilicus using cold blade morcellation within an endobag before the operation was completed. The vaginal cuff was laparoscopically sutured bi-directionally with a continuous running suture using the remainder after the ligation of both uterine vessels and then stitched at the midline. The retrieved uterus was morcellated in the same manner as described above. All patients received nonsteroidal anti-inflammatory drugs administered intravenously three times a day or patient-controlled analgesia until the second postoperative day. The intravenous patient-controlled analgesia was removed or injected analgesics were changed to oral analgesics on postoperative day 1.

### 2.3. Statistical Analysis

Continuous variables are expressed as the mean ± standard deviation (SD), and categorial variables are reported as numbers (percentages). To compare the proportions of categorical variables between the two groups, we used a chi-square test or Fisher’s exact test. For comparison of continuous variables between the SP-RM and RP-RM groups or the SP-RH and RP-RH groups, we used Student’s *t*-test or the Mann–Whitney U test. The data were normally distributed (*p* > 0.05, Kolmogorov–Smirnov test). SPSS version 21.0 (SPSS Inc., Chicago, IL, USA) was used for all statistical analyses, and *p* < 0.05 was considered statistically significant.

## 3. Results

### 3.1. Baseline Patient Characteristics

The demographic and clinical characteristics of patients are shown in Table 1. The mean age of the patients was 38.76 ± 6.96 years, and the mean body mass index was 22.83 ± 3.69 kg/m^2^. Of the 184 cases, 24 patients had a history of cesarean section, and 24 patients had a history of pelvic surgery. The patients received the following diagnoses: leiomyoma of the uterus (n = 166), adenomyosis (n = 10), endometrial intraepithelial neoplasia (n = 3), cervical carcinoma in situ (n = 1), endometrial hyperplasia (n = 1), endometrial polyp (n = 1), unicornuate uterus (n = 1), and uterine didelphys (n = 1) by pathologic confirmation.

The surgical outcomes are described in Table 2. The mean anesthesia time was 176.74 ± 56.07 min. The mean operation time was 146.68 ± 50.16 min. No case required an additional assistant port or conversion to laparotomy. The mean estimated blood loss was 172.02 ± 152.23 mL. The mean change in hemoglobin level was 2.61 ± 2.59 g/dL. Only 10 of the 184 cases (5.44%) received intraoperative or postoperative blood transfusion. The mean postoperative hospital stay was 2.8 ± 0.9 days. Approximately 3% of the patients (n = 6) showed postoperative fever within 48 h. Of the patients who underwent hysterectomy, one patient each in the SP and RP groups had a urinary tract injury and underwent urologic surgery. No other patients had a bowel injury, postoperative ileus, or unplanned emergency room visits.

### 3.2. Comparison Between Single-Port Robotic Myomectomy and Reduced-Port Robotic Myomectomy

A total of 94 patients in the SP-RM group and 38 patients in RP-RM group. Table 3 shows the detailed demographic and surgical outcomes in the women who received robotic myomectomy. There were no differences in demographics between the SP-RM group and the RP-RM group. The most common type of main myoma was intramural in both groups. And the number of leiomyomas over 3 cm, maximal diameter, and weight of removed leiomyomas were not different between the two groups. However, the number of removed leiomyoma was higher in the RP-RM group than the SP-RM group (4.26 vs. 2.70, *p* = 0.009). The rate of concomitant surgery was higher in the RP-RM group than the SP-RM group (52.6% vs. 30.9%, *p* = 0.019). There were no differences in surgical outcomes such as anesthesia time, operation time, estimated blood loss, postoperative hemoglobin change, cases of transfusion, postoperative hospital stay, and postoperative fever within 48 h between the two groups. No other special complications occurred in either group.

### 3.3. Comparison Between Single-Port Robotic Hysterectomy and Reduced-Port Robotic Hysterectomy

A total of 33 patients in the SP-RH group and 19 patients in the RP-RH group. Table 4 shows the detailed demographic and surgical outcomes in the women who received robotic myomectomy. There were no differences in demographics between the SP-RH group and the RP-RH group. The rate of concomitant surgery was slightly higher in the SP-RH group than the RP-RH group, but it was not statistically significant (75.7% vs. 47.4%, *p* = 0.1). There was no statistical difference in the total weight of the removed uteruses between the two groups. However, the total anesthesia time and operating time were longer in the SP-RH group than the RP-RH group (183.09 ± 55.17 vs. 135.74 ± 26.58, *p* = 0.001 and 159.8 ± 55.0 min vs. 113.6 ± 24.6 min, *p* = 0.001). But there were no differences in other surgical outcomes such as estimated blood loss, postoperative hemoglobin change, cases of transfusion, postoperative hospital stay, and postoperative fever within 48 h between the two groups. When comparing the SP and PR groups in the hysterectomy group, one patient in each group developed urinary tract damage, and this complication was not statistically significant between the two groups (*p* = 0.69).

## 4. Discussion

Both single-port robotic surgery using the da Vinci^®^ SP system and reduced-port robotic surgery using the da Vinci^®^ Si or Xi systems for myomectomy and hysterectomy were shown to be safe and effective surgical options with cosmetic benefits and a minimal risk of conversion to laparotomy. All the complications were classified as grade 1 and 2 according to the Dindo–Clavien classification [15].

Previous studies of robotic single-site myomectomy using the da Vinci^®^ Si or Xi systems reported that the mean operation time ranged from 131.4 to 144.5 min and that the hemoglobin change was 1.4 g/dL. The mean number and maximal diameter of the removed fibroids were 2–2.4 and 6.5 cm, respectively, in these studies [16,17,18]. In this study, the mean operation times of SP-RM and RP-RM were 150.55 min and 142.26 min, respectively. The mean hemoglobin change in SP-RM and RP-RM were 2.66 and 3.07, respectively. The mean number, mean diameter, and maximal diameter of the removed fibroids in SP-RM and RP-RM were 2.7 (range 1–11), 8.22 cm and 4.26 (range 1–21), and 8.42 cm, respectively. Compared to previous studies, the operation time was longer and the hemoglobin change was greater in this study. We believe that this difference results from the greater number of and larger myomas in our study. Previous studies on robotic single-site hysterectomy using the da Vinci^®^ Si or Xi systems reported the mean operation time as ranging from 149 to 355 min and the hemoglobin change as being 1.04 g/dL. The mean removed uterus weight was 164 g [9,10]. In this study, the mean operation times of SP-RH and RP-RH were 159.79 min and 113.58 min and the hemoglobin change in SP-RH and RP-RH was 2.28 and 2.05. The mean removed weight of uteruses in SP-RH and RP-RH were 326.67 g and 347.87 g. Compared to previous studies, the greater difference in hemoglobin is thought to be due to differences in the size of the removed uteruses. And none of the cases of myomectomy and hysterectomy were converted to laparotomy or required additional ports during surgery. Through these results, we believe that single-port robotic surgery and reduced-port robotic surgery have comparable surgical results and advantages compared to robotic single-site surgery.

Comparing SP-RM and RP-RM in our study, the number of removed leiomyoma was higher in the RP-RM group than the SP-RM group (4.26 vs. 2.70, *p* = 0.009). And the rate of concomitant surgery was higher in the RP-RM group than the SP-RM group (52.6% vs. 30.9%, *p* = 0.019). More than half of the concomitant surgeries were adnexa-related surgeries such as concomitant ovarian cyst or paratubal cyst. It can be inferred from the comparison of these results that RP-RM was often selected when the number of myomas was higher before surgery. Concomitant surgeries were more frequent in RP-RM, which may have been recognized before surgery or determined during surgery. It should also be emphasized that the operation time was not longer in RP-RM despite the higher number of myomas and the higher number of concurrent surgeries. For robotic hysterectomy, the total anesthesia time and operating time were longer in the SP-RH group than the RP-RH group (183.09 ± 55.17 vs. 135.74 ± 26.58, *p* = 0.001 and 159.8 ± 55.0 min vs. 113.6 ± 24.6 min, *p* = 0.001).

Similarly to our study results, gynecologic surgery such as myomectomy and hysterectomy using the SP system confirmed feasible surgical outcomes and safety even when compared with robotic single-site surgery. The weak point of the previous robotic single-site surgery was that the instrument was non-wristed, except for the needle driver, making it difficult to perform precise procedures at multiple angles. However, in the da Vinci SP system, all tools have two joints that can articulate over 90°, allowing practically any direction of surgical motion [19,20,21]. However, compared to the instruments used for multi-port robotic surgery, the absence of instruments such as a robotic tenaculum and the sense of decreased mechanical power of the instruments when we actually used them were considered to be limitations of the da Vinci SP system. In addition, when the intra-abdominal space is narrow due to a huge myoma or uterus, the congestion of instruments from the single port itself and the restriction of the use of additional instruments by the assistant caused inconvenience. Therefore, it is thought that the number of removed myoma in our study was higher in RP-RM than in SP-RM and the surgery time was longer in SP-RH due to these factors.

The strength of this study is that, to the best of our knowledge, this is the first study of comparing RP and SP for myomectomy and hysterectomy. We suggested a feasible surgical method of robotic surgery with minimal skin incisions without purchasing a new SP robotic system. However, the weakness of this study is that we did not directly survey patients about their satisfaction with the operative wound. In addition to that, there were no objective criteria for determining how SP and RP and decisions were made through the surgeon’s preference or consultation with the patient. Therefore, more research is needed on surgical indications such as myoma size and location.

Considering the recent rapid increase in the number of robotic surgeries around the world, it is expected that there will be more technological development in the future, and more and more options will be possible using robotic systems [22].

In conclusion, the reduced-port robotic surgery with two skin incisions using the da Vinci^®^ Si or Xi systems can be an alternative to single-port robotic surgery using the da Vinci^®^ SP system for myomectomy and hysterectomy.

## Figures and Tables

**Table 1:** Baseline and preoperative patient characteristics.

Characteristics	Myomectomy	Hysterectomy	Total
**age (years, mean ± SD)**	36.57 ± 5.20	44.33 ± 7.85	38.76 ± 6.96
**BMI (kg/m^2^, mean ± SD)**	22.64 ± 3.66	23.28 ± 3.71	22.83 ± 3.69
**gravidity, median (range)**	0 (0–6)	2 (0–6)	0 (0–6)
**parity, median (range)**	0 (0–3)	1 (0–3)	0 (0–3)
**p** **revious cesarean section history**			
0, n (%)	126 (95.45)	33 (63.46)	160 (86.96)
≥1, n (%)	6 (4.55)	19 (36.54)	24 (13.04)
**p** **revious pelvic surgery history**			
0, n (%)	122 (92.42)	38 (73.08)	160 (86.96)
≥1, n (%)	10 (7.58)	14 (26.92)	24 (13.04)
**d** **iseases**		10 (9.4)	10 (9.4)
Myoma, n (%)	132 (100%)	34 (65.39%)	166 (90.23%)
Adenomyosis, n (%)		10 (19.24%)	10 (5.44%)
Endometrial intraepithelial neoplasia, n (%)		3 (5.77%)	3 (1.63%)
Cervical carcinoma in situ n (%)		1 (1.92%)	1 (0.54%)
Endometrial hyperplasia, n (%)		1 (1.92%)	1 (0.54%)
Endometrial polyp, n (%)		1 (1.92%)	1 (0.54%)
Unicornuate uterus, n (%)		1 (1.92%)	1 (0.54%)
Uterine didelphys, n (%)		1 (1.92%)	1 (0.54%)

Abbreviations: SD, standard deviation.

**Table 2:** Surgical outcomes of patients.

Characteristics	
**a** **nesthesia time (min, mean ± SD)**	176.74 ± 56.07
**o** **peration time (min, mean ± SD)**	146.68 ± 50.16
**c** **onversion to multiport or laparotomy, n (%)**	0 (0)
**e** **stimated blood loss (mL, mean ± SD)**	172.02 ± 152.23
**p** **ostoperative hemoglobin change (g/dL, mean ± SD)**	2.61 ± 2.59
**t** **ransfusion**	
0, n (%)	174 (94.56%)
≥1, n (%)	10 (5.44%)
**postoperative hospital stay (day, mean ± SD)**	2.09 ± 0.48
**p** **ostoperative fever (within 48 h)**	
0, n (%)	178 (96.74%)
≥1, n (%)	6 (3.26%)

Abbreviations: SD, standard deviation.

**Table 3:** Baseline characteristics and surgical outcomes of robotic myomectomy (n = 132).

	SP-RM (n = 94)	RP-RM (n = 38)	*p* Value
**age (years, mean ± SD)**	36.51 ± 5.44	36.71 ± 4.60	0.842
**gravidity, median (range)**	0 (0–6)	0 (0–3)	0.261
**parity, median (range)**	0 (0–2)	0 (0–3)	0.259
**BMI (kg/m^2^, mean ± SD)**	22.42 ± 3.83	23.21 ± 3.19	0.261
**previous cesarean section history**			
0, n (%)	90 (95.7%)	36 (94.7%)	0.801
≥1, n (%)	4 (4.3%)	2 (5.3%)	
**previous pelvic surgery history**			
0, n (%)	86 (91.5%)	36 (94.7%)	0.523
≥1, n (%)	8 (8.5%)	2 (5.3%)	
**type of main myoma**			
**intramural, n (%)**	79 (84.0%)	31 (81.6%)	0.819
**submucosal, n (%)**	2 (2.1%)	2 (5.3%)	
**subserosal, n (%)**	3 (3.2%)	1 (2.6%)	
**others (intraligamentary, cervical), n (%)**	10 (10.6%)	4 (10.5%)	
**maximal myoma diameter (cm, mean ± SD)**	8.22 ± 2.36	8.42 ± 2.70	0.676
numbers of myomas, removed, median (range) *	2.70 (1–11)	4.26 (1–21)	0.009 *
**multiple myomas, n (%)**	41 (43.6)	12 (31.6)	0.201
**numbers of myomas > 3 cm, median (range)**	1.54 (1–7)	1.74 (1–4)	0.353
**weight of removed myomas (g, mean ± SD)**	218.77 ± 189.54	215.05 ± 150.23	0.914
concomitant surgery, n (%) *	29 (30.9)	20 (52.6)	0.019 *
**anesthesia time (min, mean ± SD)**	184.70 ± 64.63	172.05 ± 31.50	0.252
**operation time (min, mean ± SD)**	150.55± 55.93	142.26 ± 29.87	0.389
**estimated blood loss (mL, mean ± SD)**	188.83 ± 182.79	182.37 ± 91.84	0.836
**postoperative hemoglobin change (g/dL, mean ± SD)**	2.66 ± 1.25	3.07 ± 1.32	0.094
**transfusion**			
0, n (%)	87 (92.6)	38 (100)	0.084
≥1, n (%)	7 (7.4)	0 (0)	
**postoperative hospital stay (day, mean ± SD)**	2.04 ± 0.20	2.03 ± 0.16	0.661
**postoperative fever (within 48 h)**			
0, n (%)	90 (94.7)	36 (94.7)	0.99
≥1, n (%)	4 (5.3)	2 (5.3)	

Abbreviations: BMI, body mass index; SP-RM, single-port robotic myomectomy; RP-RM, reduced-port robotic myomectomy; SD, standard deviation. * indicates a significant difference.

**Table 4:** Baseline characteristics and surgical outcomes of robotic hysterectomy (n = 52).

	SP-RH (n = 33)	RP-RH (n = 19)	*p* Value
**age (years, mean ± SD)**	45.33 ± 7.59	42.58 ± 8.18	0.226
**gravidity, median (range)**	2 (0–6)	1 (0–3)	0.517
**parity, median (range)**	1 (0–3)	1 (0–2)	0.139
**BMI (kg/m^2^, mean ± SD)**	23.07 ± 3.32	23.66 ± 4.37	0.583
**previous cesarean section history**			
0, n (%)	20 (60.6)	13 (68.4)	0.34
≥1, n (%)	13 (39.4)	6 (31.6)	
**previous pelvic surgery history**			
0, n (%)	25 (75.8)	13 (68.4)	0.566
≥1, n (%)	8 (24.2)	6 (31.6)	
**weight of uterus (g, mean ± SD)**	326.67± 230.76	347.87± 271.75	0.766
**concomitant surgery, n (%)**	25 (75.7)	9 (47.4)	0.1
anesthesia time (min, mean ± SD) *	183.09 ± 55.17	135.74 ± 26.58	0.001 *
operation time (min, mean ± SD) *	159.79 ± 55.04	113.58 ± 24.61	0.001 *
**estimated blood loss (mL, mean ± SD)**	153.33 ± 131.69	94.21 ± 74.86	0.079
**postoperative hemoglobin change (g/dL, mean ± SD)**	2.28 ± 1.30	2.05 ± 1.06	0.506
**transfusion**			
0, n (%)	30 (90.9)	19 (100)	0.176
≥1, n (%)	3 (9.1)	0 (0)	
**postoperative hospital stay (day, mean ± SD)**	2.19 ± 0.90	2.26 ± 0.81	0.764
**postoperative fever (within 48 h)**			
0, n (%)	33 (100)	18 (94.7)	0.183
≥1, n (%)	0 (0)	1 (5.3)	

Abbreviations: BMI, body mass index; SP-RH, single-port robotic hysterectomy. RP-RH, reduced-port robotic hysterectomy; SD, standard deviation. * indicates a significant difference.

## Data Availability

The excel data used to support the findings of this study were supplied by Sa Ra Lee under license, and requests for access to these data should be made to Sa Ra Lee, leesr@amc.seoul.kr.
